# Associations between anemia and FGF23 in the CKiD study

**DOI:** 10.1007/s00467-023-06160-0

**Published:** 2023-09-26

**Authors:** Elizabeth Thomas, Alexandra M. Klomhaus, Marciana L. Laster, Susan L. Furth, Bradley A. Warady, Isidro B. Salusky, Mark R. Hanudel

**Affiliations:** 1 Department of Pediatrics, David Geffen School of Medicine at UCLA, Los Angeles, CA USA; 2 Department of Medicine, David Geffen School of Medicine at UCLA, Los Angeles, CA USA; 3 Department of Pediatrics, Children’s Hospital of Philadelphia, Philadelphia, PA USA; 4 Department of Pediatrics, Children’s Mercy Kansas City, Kansas City, MO USA

**Keywords:** Pediatrics, Chronic kidney disease, Fibroblast growth factor 23, Anemia, Iron

## Abstract

**Background:**

Fibroblast growth factor 23 (FGF23) is a bone-derived hormone that plays a central role in chronic kidney disease-mineral bone disorder and is associated with CKD progression and cardiovascular morbidity. Factors related to CKD-associated anemia, including iron deficiency, can increase FGF23 production. This study aimed to assess whether anemia and/or iron deficiency are associated with increased circulating concentrations of FGF23 in the large, well-characterized Chronic Kidney Disease in Children (CKiD) study cohort.

**Methods:**

Hemoglobin concentrations, iron parameters, C-terminal (total) FGF23, intact FGF23, and relevant covariables were measured in cross-sectional analysis of CKiD study subjects.

**Results:**

In 493 pediatric patients with CKD (median [interquartile range] age 13 [9, 16] years), the median estimated glomerular filtration rate was 48 [35, 61] ml/min/1.73 m^2^, and 103 patients (21%) were anemic. Anemic subjects had higher total FGF23 concentrations than non-anemic subjects (204 [124, 390] vs. 109 [77, 168] RU/ml, *p* < 0.001). In multivariable linear regression modeling, anemia was independently associated with higher total FGF23, after adjustment for demographic, kidney-related, mineral metabolism, and inflammatory covariables (standardized β (95% confidence interval) 0.10 (0.04, 0.17), *p* = 0.002). In the subset of subjects with available iron parameters (n = 191), iron deficiency was not associated with significantly higher total FGF23 concentrations. In the subgroup that had measurements of both total and intact FGF23 (n = 185), in fully adjusted models, anemia was significantly associated with higher total FGF23 (standardized β (95% CI) 0.16 (0.04, 0.27), *p* = 0.008) but not intact FGF23 (standardized β (95% CI) 0.02 (−0.12, 0.15), *p* = 0.81).

**Conclusions:**

In this cohort of pediatric patients with CKD, anemia was associated with increased total FGF23 levels but was not independently associated with elevated intact FGF23, suggesting possible effects on both FGF23 production and cleavage. Further studies are warranted to investigate non-mineral factors affecting FGF23 production and metabolism in CKD.

**Graphical abstract:**

**Figure:**
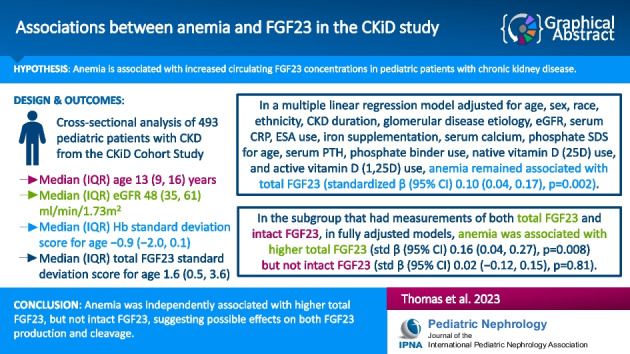
A higher resolution version of the Graphical abstract is available as Supplementary information

**Supplementary Information:**

The online version contains supplementary material available at 10.1007/s00467-023-06160-0.

## Introduction

Fibroblast growth factor 23 (FGF23) is an important hormone implicated in the pathogenesis of chronic kidney disease-mineral bone disorder (CKD-MBD). FGF23 is secreted predominantly by osteocytes to regulate phosphate and 1,25-dihydroxyvitamin D. In the early stages of CKD, bone [1] and circulating [2–5] FGF23 levels increase, and continue to rise as CKD progresses and glomerular filtration rate declines. Although increases in FGF23 help to maintain normophosphatemia until late in the course of CKD [2, 5], FGF23-induced suppression of 1,25-dihydroxyvitamin D contributes to secondary hyperparathyroidism.

Moreover, high levels of FGF23 have been associated with various adverse events in patients with CKD. In adult [6, 7] and pediatric [8] patients with CKD, higher circulating concentrations of FGF23 are associated with disease progression. Additionally, elevated FGF23 levels in CKD may contribute to cardiovascular morbidity. Pre-clinical in vitro and in vivo studies have demonstrated that FGF23 can directly induce cardiomyocyte hypertrophy [9]. In adult [9, 10] and pediatric [11] patients with CKD, higher circulating concentrations of FGF23 are associated with increased left ventricular mass. Lastly, in CKD, elevated FGF23 levels are associated with impaired neutrophil activation [12] and infection-related morbidity [13]. Potentially due to these multisystemic adverse effects, higher FGF23 levels in CKD are independently associated with increased overall mortality rates [7, 9, 10, 14].

In the setting of CKD, various factors may contribute to increased FGF23 levels. Mineral metabolism factors, including phosphate and 1,25-dihydroxyvitamin D, are well-known stimuli of FGF23 production. Recently, however, novel, non-mineral stimuli of FGF23 production have been identified [15]. Specifically, anemia-related factors have been shown to increase FGF23 production, including iron deficiency [16–19], which is common in adult [20] and pediatric [21] CKD, and increased erythropoietin [22–27], levels of which increase in response to decreasing hemoglobin concentrations in mild–moderate CKD [28]. In the present study, we sought to evaluate how contributory anemia is to elevated FGF23 levels in a cohort of pediatric patients with CKD, hypothesizing that anemia is associated with increased circulating FGF23 concentrations.

## Methods

### Study design and participants

A retrospective, observational, cross-sectional study was performed which included 493 pediatric subjects from the multicenter Chronic Kidney Disease in Children (CKiD) Cohort Study. The CKiD study is an observational cohort study of children aged 6 months to 16 years with CKD. Enrollment began in January 2005. Complete details of the CKiD study design and methods have been published [29]. The CKiD study was approved by the institutional review board of each participating institution. Written informed consent and assent, when appropriate, was obtained from all parents/legal guardians of the enrolled study subjects.

### Predictor variable

The predictor variable was the presence vs. absence of anemia, as defined by hemoglobin concentration thresholds listed in the 2012 KDIGO guidelines for pediatric patients with CKD (Supplemental Table 1) [30], independent of erythropoiesis-stimulating agent (ESA) usage. Also, given that normal hemoglobin concentrations vary by age within the pediatric population, we used published normative data to calculate hemoglobin standard deviation scores (SDS) for age [31].

### Outcome variables

The outcome variable of interest was plasma FGF23, as measured with the C-terminal (total) FGF23 ELISA kit (Immutopics) and obtained concurrently with hemoglobin concentrations. Due to non-normal data distribution, FGF23 concentrations were log-transformed prior to analysis. Also, given that normal concentrations of total FGF23 vary by age within the pediatric population, we used published normative data to calculate total FGF23 standard deviation scores (SDS) for age [32]. A subset of patients also had FGF23 measured with the intact FGF23 ELISA kit (Immutopics). Whereas the total FGF23 assay detects both full-length, intact FGF23 and C-terminal FGF23 proteolytic fragments, the intact FGF23 assay detects only the full-length form (Supplemental Fig. 1).

### Covariables

Covariables were obtained concurrently with the predictor and outcome variables, and included demographic parameters (age, sex, race, ethnicity); kidney-related factors (CKD duration, glomerular vs. non-glomerular etiology of CKD, estimated glomerular filtration rate (eGFR, calculated using the CKiD under 25 (U25) GFR estimating equations [33])); C-reactive protein (CRP); ESA usage; iron supplementation; mineral metabolism parameters (serum calcium, phosphate, and parathyroid hormone (PTH); use of phosphate binder medications, use of native vitamin D (25D), and use of active vitamin D (1,25D)); and the presence vs. absence of iron deficiency. Given that normal concentrations of serum phosphate vary by age within the pediatric population, we used published normative data to calculate phosphate standard deviation scores (SDS) for age [34]. Measures of iron status, available in a subset of patients, included serum iron, total iron binding capacity (TIBC), transferrin saturation (TSAT), and ferritin. Consistent with the 2012 KDIGO guidelines for pediatric patients with CKD, iron deficiency was defined as TSAT ≤ 20% [30]. A concurrent serum ferritin concentration of ≤ 100 ng/ml or > 100 ng/ml defined absolute iron deficiency and “functional” iron deficiency, respectively.

### Statistical analysis

A cross-sectional analysis was performed that included 493 subjects who had both hemoglobin and plasma total FGF23 measured concurrently. Descriptive statistics are expressed as numbers and percentages for categorical variables, and as medians and interquartile ranges (IQR) for continuous variables. Comparisons between groups were made using Chi-square tests (or Fisher exact tests, when appropriate) and Mann–Whitney U tests, for categorical and continuous variables, respectively. Univariable and multivariable linear regression analysis was used to evaluate associations between independent variables, including the presence vs. absence of anemia, and the dependent variable of log-transformed total FGF23. GraphPad Prism 10.0.1 was used for statistical analysis, with *P*-values < 0.05 considered statistically significant.

## Results

### Cohort characteristics

The cohort included 493 pediatric patients with CKD, with a median (IQR) age of 13 (9, 16) years and a median eGFR of 48 (35, 61) ml/min/1.73 m^2^ (Table 1). The median hemoglobin concentration was 13.1 (12.1, 14.1) g/dl, with a median hemoglobin SDS for age of −0.9 (−2.0, 0.1). The median C-terminal (total) FGF23 concentration was 118 (82, 204) RU/ml, with a median total FGF23 SDS for age of 1.6 (0.5, 3.6).

**Table 1:** Demographic data, clinical characteristics, and biochemical parameters for patients from the Chronic Kidney Disease in Children (CKiD) Cohort Study

Variable	n	N (%) / Median [IQR]
Age (years)	493	13 [9, 16]
Sex (male)	493	311 (63%)
Race:	493	
White		342 (69%)
African American		67 (14%)
Asian		11 (2%)
Native American		9 (2%)
Other		19 (4%)
More than one race		45 (9%)
Hispanic ethnicity	493	56 (11%)
Etiology of CKD:	493	
Non-glomerular		378 (77%)
Glomerular		115 (23%)
CKD duration (years)	487	10 [6, 14]
Height SDS for age	480	−0.5 [−1.3, 0.2]
Weight SDS for age	492	−0.1 [−0.8, 0.9]
Creatinine (mg/dl)	493	1.20 [0.90, 1.70]
Estimated GFR (ml/min/1.73 m^2^)	492	48 [35, 61]
Hemoglobin (g/dl)	493	13.1 [12.1, 14.1]
Hemoglobin SDS for age	493	−0.9 [−2.0, 0.1]
Anemia	493	103 (21%)
MCV (fl)	493	84.2 [81.0, 87.4]
RDW (%)	466	13.0 [12.4, 13.5]
Iron (μg/dl)	191	80 [57, 101]
TIBC (μg/dl)	192	310 [289, 339]
TSAT (%)	191	25 [18, 33]
Ferritin (ng/ml)	190	44 [25, 83]
Iron supplementation	493	148 (30%)
ESA use	493	53 (11%)
Phosphate binder use	493	84 (17%)
Native vitamin D (25D) use	493	76 (15%)
Active vitamin D (1,25D) use	493	165 (33%)
C-reactive protein (mg/l)	478	0.3 [0.2, 1.4]
Albumin (g/dl)	489	4.5 [4.2, 4.7]
Calcium (mg/dl)	489	9.6 [9.3, 9.9]
Phosphate (mg/dl)	488	4.4 [3.9, 5.0]
Phosphate SDS for age	488	−0.8 [−1.5, 0.0]
Parathyroid hormone (pg/ml)	472	51 [32, 80]
C-terminal (total) FGF23 (RU/ml)	493	118 [82, 204]
C-terminal (total) FGF23 SDS for age	493	1.6 [0.5, 3.6]
Intact FGF23 (pg/ml)	185	65 [44, 113]

CKD: chronic kidney disease, ESA: erythropoiesis-stimulating agent, FGF23: fibroblast growth factor 23, GFR: glomerular filtration rate, MCV: mean corpuscular volume, RDW: red cell distribution width, RU: relative units, SDS: standard deviation score, TIBC: total iron binding capacity, TSAT: transferrin saturation

### Study subjects with and without anemia

In this cohort, 103 participants (21%) were anemic. Compared to subjects without anemia, those with anemia had a lower median (IQR) eGFR (32 [21, 44] vs. 51 [40, 65] ml/min/1.73 m^2^, *p* < 0.001), a higher median phosphate SDS for age (−0.5 [−1.3, 0.7] vs. −0.8 [−1.5, −0.2], *p* < 0.001), a higher median total FGF23 concentration (204 [124, 390] vs. 109 [77, 168] RU/ml, *p* < 0.001), and a higher median total FGF23 SDS for age (3.5 [1.6, 6.6] vs. 1.3 [0.4, 2.8], *p* < 0.001) (Table 2). Across CKD stages and eGFR quartiles, anemic subjects generally had higher total FGF23 levels than non-anemic subjects, although statistical significance was not reached in all stages or quartiles (Supplemental Table 2).

**Table 2:** Demographic data, clinical characteristics, and biochemical parameters stratified by the absence vs. presence of anemia

Variable	n	No Anemia	n	Anemia	*P*-value
Age (years)	390	12 [8, 16]	103	14 [12, 16]	0.005
Sex (male)	390	252 (65%)	103	59 (57%)	0.17
Race:	390		103		0.17
White		271 (69%)		71 (69%)	
African American		54 (14%)		13 (13%)	
Asian		9 (2%)		2 (2%)	
Native American		6 (2%)		3 (3%)	
Other		11 (3%)		8 (8%)	
More than one race		39 (10%)		6 (6%)	
Hispanic ethnicity	390	38 (10%)	103	18 (17%)	0.036
Etiology of CKD:	390		103		0.013
Non-glomerular		309 (79%)		69 (67%)	
Glomerular		81 (21%)		34 (33%)	
CKD duration (years)	386	10 [6, 14]	101	11 [7, 15]	0.20
Height SDS for age	382	−0.5 [−1.3, 0.3]	103	−3.1 [−4.1, −2.5]	< 0.001
Weight SDS for age	389	0.0 [−0.7, 0.9]	103	−0.3 [−1.5, 0.7]	0.017
Creatinine (mg/dl)	390	1.10 [0.84, 1.48]	103	1.83 [1.30, 2.89]	< 0.001
Estimated GFR (ml/min/1.73 m^2^)	389	51 [40, 65]	103	32 [21, 44]	< 0.001
Hemoglobin (g/dl)	390	13.5 [12.7, 14.3]	103	11.3 [10.5, 11.8]	< 0.001
Hemoglobin SDS for age	390	−0.5 [−1.3, 0.4]	103	−3.1 [−4.1, −2.5]	< 0.001
MCV (fl)	390	84.1 [81.0, 87.2]	103	84.2 [81.1, 88.0]	0.28
RDW (%)	371	12.9 [12.4, 13.5]	95	13.2 [12.7, 13.9]	< 0.001
Iron (μg/dl)	148	85 [59, 102]	43	72 [48, 88]	0.046
TIBC (μg/dl)	148	316 [294, 340]	44	296 [273, 314]	0.001
TSAT (%)	148	26 [18, 34]	43	24 [16, 33]	0.59
Ferritin (ng/ml)	146	40 [24, 70]	44	70 [31, 139]	0.003
Iron supplementation	390	96 (25%)	103	52 (50%)	< 0.001
ESA use	390	26 (7%)	103	27 (26%)	< 0.001
Phosphate binder use	390	51 (13%)	103	33 (32%)	< 0.001
Native vitamin D (25D) use	390	55 (14%)	103	21 (20%)	0.12
Active vitamin D (1,25D) use	390	106 (27%)	103	59 (57%)	< 0.001
C-reactive protein (mg/l)	379	0.4 [0.2, 1.4]	99	0.3 [0.1, 1.6]	0.27
Albumin (g/dl)	387	4.5 [4.3, 4.7]	102	4.4 [4.1, 4.6]	< 0.001
Calcium (mg/dl)	387	9.6 [9.4, 9.9]	102	9.4 [9.0, 9.7]	< 0.001
Phosphate (mg/dl)	387	4.4 [3.9, 5.0]	101	4.6 [4.0, 5.2]	0.07
Phosphate SDS for age	387	−0.8 [−1.5, −0.2]	101	−0.5 [−1.3, 0.7]	< 0.001
Parathyroid hormone (pg/ml)	376	49 [31, 71]	96	79 [40, 169]	< 0.001
C-terminal (total) FGF23 (RU/ml)	390	109 [77, 168]	103	204 [124, 390]	< 0.001
C-terminal (total) FGF23 SDS for age	390	1.3 [0.4, 2.8]	103	3.5 [1.6, 6.6]	< 0.001
Intact FGF23 (pg/ml)	154	64 [42, 111]	31	74 [54, 176]	0.012

Data presented as numbers and percentages, or as medians and interquartile ranges. CKD: chronic kidney disease, ESA: erythropoiesis-stimulating agent, FGF23: fibroblast growth factor 23, GFR: glomerular filtration rate, MCV: mean corpuscular volume, RDW: red cell distribution width, RU: relative units, SDS: standard deviation score, TIBC: total iron binding capacity, TSAT: transferrin saturation

### Study subjects with and without ESA use

In this cohort, 53 participants (11%) were treated with ESAs. Compared to untreated subjects, those receiving ESAs had a lower median (IQR) eGFR (29 [20, 39] vs. 50 [38, 63] ml/min/1.73 m^2^, *p* < 0.001), a higher prevalence of anemia (51% vs. 17%, *p* < 0.001), a higher median total FGF23 concentration (254 [129, 459] vs. 113 [79, 184] RU/ml, *p* < 0.001), and a higher median total FGF23 SDS for age (4.3 [1.9, 7.2] vs. 1.4 [0.4, 3.4], *p* < 0.001) (Table 3).

**Table 3:** Demographic data, clinical characteristics, and biochemical parameters stratified by ESA non-use vs. use

Variable	n	No ESA Use	n	ESA Use	*P*-value
Age (years)	440	13 [9, 16]	53	14 [10, 16]	0.99
Sex (male)	440	282 (64%)	53	29 (55%)	0.23
Race:	440		53		0.43
White		304 (69%)		38 (72%)	
African American		61 (14%)		6 (11%)	
Asian		9 (2%)		2 (4%)	
Native American		8 (2%)		1 (2%)	
Other		15 (3%)		4 (8%)	
More than one race		43 (10%)		2 (4%)	
Hispanic ethnicity	440	47 (11%)	53	9 (17%)	0.17
Etiology of CKD:	440		53		0.005
Non-glomerular		346 (79%)		32 (60%)	
Glomerular		94 (21%)		21 (40%)	
CKD duration (years)	435	10 [6, 14]	52	9 [4, 14]	0.11
Height SDS for age	428	−0.5 [−1.3, 0.2]	52	−1.1 [−1.7, −0.3]	0.002
Weight SDS for age	439	0.0 [−0.7, 1.0]	53	−0.4 [−1.5, 0.1]	0.001
Creatinine (mg/dl)	440	1.18 [0.89, 1.56]	53	1.90 [1.50, 2.75]	< 0.001
Estimated GFR (ml/min/1.73 m^2^)	439	50 [38, 63]	53	29 [20, 39]	< 0.001
Hemoglobin (g/dl)	440	13.3 [12.2, 14.2]	53	11.8 [11.2, 12.7]	< 0.001
Hemoglobin SDS for age	440	−0.8 [−1.9, 0.1]	53	−2.2 [−3.9, −1.3]	< 0.001
Anemia	440	76 (17%)	53	27 (51%)	< 0.001
MCV (fl)	440	84.0 [81.0, 87.0]	53	86.8 [82.9, 90.4]	0.004
RDW (%)	416	12.9 [12.4, 13.5]	50	13.6 [12.7, 15.2]	< 0.001
Iron (μg/dl)	167	79 [57, 101]	24	84 [57, 104]	0.76
TIBC (μg/dl)	168	314 [293, 340]	24	286 [248, 309]	< 0.001
TSAT (%)	167	25 [18, 32]	24	31 [19, 39]	0.08
Ferritin (ng/ml)	166	39 [24, 70]	24	107 [59, 329]	< 0.001
Iron supplementation	440	103 (23%)	53	45 (85%)	< 0.001
Phosphate binder use	440	62 (14%)	53	22 (42%)	< 0.001
Native vitamin D (25D) use	440	71 (16%)	53	5 (9%)	0.23
Active vitamin D (1,25D) use	440	128 (29%)	53	37 (70%)	< 0.001
C-reactive protein (mg/l)	427	0.4 [0.2, 1.4]	51	0.2 [0.03, 2.4]	0.08
Albumin (g/dl)	436	4.5 [4.3, 4.7]	53	4.4 [4.1, 4.7]	0.20
Calcium (mg/dl)	436	9.6 [9.3, 9.9]	53	9.5 [9.2, 9.8]	0.14
Phosphate (mg/dl)	436	4.4 [3.9, 4.9]	52	5.1 [4.2, 5.5]	< 0.001
Phosphate SDS for age	436	−0.8 [−1.5, −0.2]	52	0.3 [−1.0, 1.0]	< 0.001
Parathyroid hormone (pg/ml)	421	50 [32, 75]	51	80 [47, 190]	< 0.001
C-terminal (total) FGF23 (RU/ml)	440	113 [79, 184]	53	254 [129, 459]	< 0.001
C-terminal (total) FGF23 SDS for age	440	1.4 [0.4, 3.4]	53	4.3 [1.9, 7.2]	< 0.001
Intact FGF23 (pg/ml)	179	65 [44, 113]	6	82 [45, 155]	0.66

Data presented as numbers and percentages, or as medians and interquartile ranges. CKD: chronic kidney disease, ESA: erythropoiesis-stimulating agent, FGF23: fibroblast growth factor 23, GFR: glomerular filtration rate, MCV: mean corpuscular volume, RDW: red cell distribution width, RU: relative units, SDS: standard deviation score, TIBC: total iron binding capacity, TSAT: transferrin saturation

### Study subjects with and without iron deficiency

A subset of the cohort (n = 191) had iron parameters measured (Table 4). In this subset, 67 participants (35%) were iron deficient, as defined by a TSAT ≤ 20%. Almost all of the iron deficient subjects (91%) had absolute iron deficiency, as defined by TSAT ≤ 20% and serum ferritin ≤ 100 ng/ml. Compared to subjects without iron deficiency, those with iron deficiency had a lower median (IQR) hemoglobin SDS for age (−1.4 [−2.4, −0.5] vs. −0.7 [−1.8, 0.4], *p* = 0.009), although the prevalence of anemia was similar in the two groups (27% vs. 20%, *p* = 0.36). Total FGF23 concentrations (134 [91, 207] vs. 126 [88, 196], *p* = 0.55) and total FGF23 SDS for age (2.1 [0.7, 3.9] vs. 1.9 [0.7, 3.7], *p* = 0.62) did not differ between the iron deficient and sufficient groups. Across CKD stages and eGFR quartiles, iron deficiency was not associated with higher total FGF23 in any stage or quartile (Supplemental Table 3).

**Table 4:** Demographic data, clinical characteristics, and biochemical parameters stratified by iron sufficiency vs. deficiency

Variable	n	No Iron Deficiency	n	Iron Deficiency	*P*-value
Age (years)	124	13 [8, 16]	67	12 [8, 16]	0.52
Sex (male)	124	81 (65%)	67	41 (61%)	0.64
Race:	124		67		0.68
White		84 (68%)		44 (66%)	
African American		21 (17%)		9 (13%)	
Asian		2 (2%)		3 (4%)	
Native American		2 (2%)		1 (1%)	
Other		4 (3%)		1 (1%)	
More than one race		11 (9%)		9 (13%)	
Hispanic ethnicity	124	7 (6%)	67	8 (12%)	0.16
Etiology of CKD:	124		67		0.14
Non-glomerular		94 (76%)		57 (85%)	
Glomerular		30 (24%)		10 (15%)	
CKD duration (years)	123	10 [6, 15]	67	10 [6, 13]	0.77
Height SDS for age	120	−0.7 [−1.3, 0.1]	66	−0.5 [−1.4, 0.2]	0.98
Weight SDS for age	123	−0.2 [−0.9, 0.6]	67	0.1 [−0.9, 1.0]	0.21
Creatinine (mg/dl)	124	1.30 [0.91, 1.80]	67	1.29 [1.00, 1.80]	0.90
Estimated GFR (ml/min/1.73 m^2^)	124	46 [33, 56]	67	44 [34, 54]	0.58
Hemoglobin (g/dl)	124	13.3 [12.0, 14.3]	67	12.6 [11.7, 13.4]	0.012
Hemoglobin SDS for age	124	−0.7 [−1.8, 0.4]	67	−1.4 [−2.4, −0.5]	0.009
Anemia	124	25 (20%)	67	18 (27%)	0.36
MCV (fl)	124	84.2 [81.2, 87.1]	67	83.3 [79.6, 85.6]	0.037
RDW (%)	112	13.0 [12.5, 13.6]	61	13.3 [12.7, 14.2]	0.035
Iron (μg/dl)	124	93 [80, 110]	67	44 [37, 59]	< 0.001
TIBC (μg/dl)	124	308 [280, 333]	67	326 [299, 353]	0.002
TSAT (%)	124	31 [26, 36]	67	15 [10, 18]	< 0.001
Ferritin (ng/ml)	123	47 [27, 95]	66	35 [18, 72]	0.003
Type of Iron Deficiency:	n/a		66		n/a
Absolute Iron Deficiency		n/a		60 (91%)	
Functional Iron Deficiency		n/a		6 (9%)	
Iron supplementation	124	37 (30%)	67	25 (37%)	0.33
ESA use	124	17 (14%)	67	7 (10%)	0.65
Phosphate binder use	124	28 (23%)	67	6 (9%)	0.018
Native vitamin D (25D) use	124	13 (10%)	67	4 (6%)	0.43
Active vitamin D (1,25D) use	124	46 (37%)	67	22 (33%)	0.64
C-reactive protein (mg/l)	118	0.2 [0.04, 0.6]	65	0.7 [0.3, 3.0]	< 0.001
Albumin (g/dl)	124	4.5 [4.3, 4.7]	67	4.5 [4.3, 4.8]	0.73
Calcium (mg/dl)	124	9.7 [9.4, 10.1]	67	9.7 [9.5, 9.9]	0.90
Phosphate (mg/dl)	123	4.4 [3.9, 5.0]	67	4.6 [4.2, 5.2]	0.07
Phosphate SDS for age	123	−0.8 [−1.7, −0.2]	67	−0.5 [−1.3, 0.3]	0.05
Parathyroid hormone (pg/ml)	115	53 [33, 103]	65	62 [40, 105]	0.26
C-terminal (total) FGF23 (RU/ml)	124	126 [88, 196]	67	134 [91, 207]	0.55
C-terminal (total) FGF23 SDS for age	124	1.9 [0.7, 3.7]	67	2.1 [0.7, 3.9]	0.62

Data presented as numbers and percentages, or as medians and interquartile ranges. CKD: chronic kidney disease, ESA: erythropoiesis-stimulating agent, FGF23: fibroblast growth factor 23, GFR: glomerular filtration rate, MCV: mean corpuscular volume, RDW: red cell distribution width, RU: relative units, SDS: standard deviation score, TIBC: total iron binding capacity, TSAT: transferrin saturation

### Relationship between anemia and total FGF23

Multiple linear regression modeling was performed to evaluate the association between anemia and total FGF23. In models adjusted for demographic parameters, kidney-related factors, inflammation, ESA use, and mineral metabolism parameters, anemia was significantly associated with higher total FGF23 values (standardized β (95% confidence interval (CI)) 0.10 (0.04, 0.17), *p* = 0.002, n = 459 in the fully adjusted model; Table 5, Supplemental Table 4). In the subset of subjects with available iron parameters, upon further adjustment for iron deficiency, the association between anemia and total FGF23 was attenuated (standardized β (95% CI) 0.10 (−0.02, 0.21), *p* = 0.09, n = 177 in the fully adjusted model; Supplemental Table 5).

**Table 5:** Associations between anemia and log-transformed C-terminal (total) fibroblast growth factor 23 for patients from the Chronic Kidney Disease in Children (CKiD) Cohort Study

Model	Covariables	Number of Subjects	Standardized β (95% CI)	*P*-value
1	Unadjusted	493	0.29 (0.22, 0.35)	< 0.001
2	Model 1 + adjustment for age, sex, race, and ethnicity	493	0.29 (0.22, 0.35)	< 0.001
3	Model 2 + adjustment for CKD duration, glomerular disease etiology, and eGFR	486	0.13 (0.07, 0.20)	< 0.001
4	Model 3 + adjustment for CRP, ESA use, and iron supplementation	471	0.13 (0.07, 0.20)	< 0.001
5	Model 4 + adjustment for calcium, phosphate SDS for age, PTH, phosphate binder use, 25D use, and 1,25D use	459	0.10 (0.04, 0.17)	0.002

CI: confidence interval, CKD: chronic kidney disease, CRP: C-reactive protein, eGFR: estimated glomerular filtration rate, ESA: erythropoiesis-stimulating agent, PTH: parathyroid hormone, SDS: standard deviation score

### Relationship between anemia and intact FGF23

A subset of the cohort (n = 185) had plasma intact FGF23 measured, with a median concentration of 65 (44, 113) pg/ml (Table 1). Anemic subjects had a higher median intact FGF23 concentration (74 [54, 176] vs. 64 [42, 111] pg/ml, *p* = 0.012) (Table 2). However, in multiple linear regression modeling, anemia was not independently associated with intact FGF23 (Supplemental Table 6).

### Comparison of effects on total and intact FGF23

In the 185 study participants with both total and intact FGF23 measurements, in unadjusted analyses, the magnitude of the association between anemia and total FGF23 was more pronounced than the magnitude of the association between anemia and intact FGF23 (Supplemental Table 7). In adjusted analyses, anemia remained significantly associated with total FGF23, but not intact FGF23.

## Discussion

In adult [2–4] and pediatric [5] CKD, levels of FGF23 are greatly increased, which contribute to CKD-MBD pathogenesis and have been associated with multisystemic co-morbidity and mortality [6–14]. Several mineral and non-mineral factors stimulate FGF23 production. Interestingly, anemia-related factors, including iron deficiency [16–19] and erythropoietin [22–27], have been shown to increase FGF23 production. In the present study, we evaluated associations between anemia and FGF23 in a large cohort of pediatric patients with CKD, hypothesizing that anemia is associated with increased circulating FGF23 concentrations.

In the cohort evaluated, the median baseline eGFR was 48 ml/min/1.73 m^2^, consistent with CKD stage 3a. Overall, 21% of study participants were anemic, similar to what was observed in the National Health and Nutrition Examination Survey (NHANES) of adults with CKD stage 3 [35, 36]. The median (IQR) C-terminal (total) FGF23 concentration was 118 (82, 204) RU/ml, which is similar to what was observed in the large adult Chronic Renal Insufficiency Cohort (CRIC) study in subjects with eGFR 40–49 ml/min/1.73 m^2^ (135 [94,188] RU/ml), measured with the same C-terminal (total) FGF23 assay [2]. In our cohort, 43% had a total FGF23 SDS for age > 2.0 (> 2 standard deviations above the mean value for age). A similar interpretation of intact FGF23 concentrations in our cohort is not possible, given a lack of assay-specific reference values for healthy children [37].

In cross-sectional analysis, we observed that study subjects with anemia had higher circulating concentrations of total FGF23. This association remained significant after adjustment for demographic parameters, CKD-specific factors, inflammation, ESA use, iron supplementation, and mineral metabolism parameters, suggesting a robust association between anemia and higher total FGF23 values. In the subset of subjects with available iron parameters, upon further adjustment for the presence vs. absence of iron deficiency, the association between anemia and total FGF23 was attenuated. This observation could suggest that iron deficiency, which is associated with both anemia and elevated total FGF23 concentrations in patients with CKD [38, 39], confounded the association between anemia and increased total FGF23. However, in the subgroup of our cohort with iron parameters, those with iron deficiency did not have a higher prevalence of anemia or higher total FGF23 concentrations, arguing against a high degree of confounding. Alternatively, the loss of statistical significance with the addition of iron deficiency to the model was likely contributed to by the large decrease in sample size, as only a relatively small subset of the cohort (39%) had iron parameters available.

In this small subset of our cohort, iron deficiency was not associated with higher total FGF23 concentrations; however, in larger adult CKD cohorts [38, 39], this association has been observed. In adults with CKD, multivariable linear regression modeling has shown independent, inverse associations between both serum ferritin and total FGF23 levels, and between hemoglobin and total FGF23 levels [27], suggesting that both iron status and hemoglobin concentration may affect FGF23 in CKD.

Additionally, other erythropoiesis-related factors may also affect FGF23 production in CKD. Exogenous erythropoietin administration can increase total FGF23 concentrations in patients with CKD [40], so it is possible that ESA use may have at least partially mediated the association we observed between anemia and elevated total FGF23 concentrations. In our cohort, study subjects with anemia had a higher prevalence of ESA use, and ESA use was associated with higher concentrations of total FGF23. However, in adjusted models, anemia was not significantly associated with ESA use (data not shown), ESA use was not significantly associated with FGF23, and the inclusion vs. exclusion of ESA use as an independent variable did not reduce the effect of anemia on FGF23, suggesting relatively little mediating effect of ESA use.

In our multiple linear regression modeling, we presented standardized coefficients, allowing for comparison of the relative effects of different independent variables on the dependent variable (log-transformed total FGF23). In our fully adjusted model, the magnitude of the effect of anemia on total FGF23 was similar to that of eGFR, and was greater than that of phosphate SDS for age, serum PTH, and active vitamin D use. In this model, the variance inflation factors (VIF) for the mineral metabolism variables were low, suggesting little collinearity among these independent variables that could affect the coefficients. Therefore, our modeling suggests that, in this CKD cohort, anemia may be as contributory to total FGF23 concentrations as kidney function and mineral metabolism parameters.

A subset of study participants (38%) had both total and intact FGF23 measurements available. In adjusted analyses, anemia was not associated with intact FGF23. Given that differences in sample size may have contributed to the differential observations for intact FGF23 (subset) vs. total FGF23 (full cohort), we performed analyses limited to this subgroup, in which both intact and total FGF23 were measured. In this group, in adjusted analyses, anemia was associated with total FGF23, but not intact FGF23.

Associations with total FGF23 but not intact FGF23 suggest effects on FGF23 cleavage. Translated FGF23 protein can be cleaved intracellularly, resulting in secretion into the circulation of predominantly intact FGF23, predominantly C-terminal and N-terminal FGF23 fragments, or a combination of intact and fragmented FGF23 [41]. Factors that increase FGF23 transcription or translation without affecting post-translational cleavage would be expected to increase circulating concentrations of both total and intact FGF23. Conversely, factors that increase FGF23 transcription or translation while concurrently increasing post-translational cleavage would be expected to increase circulating concentrations of total FGF23 to a greater degree than circulating concentrations of intact FGF23, reflecting the presence of increased FGF23 fragments. Factors that have been reported to concurrently increase FGF23 transcription and post-translational cleavage, resulting in the disproportionate cellular secretion of FGF23 fragments, include iron deficiency [16–19], erythropoietin [22–27], and inflammation [18].

Therefore, our observation of an association between anemia and total FGF23 but not anemia and intact FGF23 suggests the effects of a factor associated with anemia that couples increased FGF23 transcription/translation with increased proteolytic cleavage. All three aforementioned factors—iron deficiency [20], increased serum erythropoietin concentrations [28], and inflammation [42]—are associated with anemia in CKD and have similar effects on FGF23 production and metabolism. Therefore, these factors may have contributed to the observed results from our multiple linear regression modeling evaluating associations between anemia and FGF23 moieties in this subgroup. However, in our final model, which included adjustments for ESA use and C-reactive protein, anemia remained associated with total FGF23 but not intact FGF23, suggesting that neither of these factors markedly confounded our observations. As the subsets of subjects with available iron parameters and intact FGF23 measurements were almost completely mutually exclusive, we could not directly evaluate the effects of iron deficiency on relationships between anemia and FGF23 moieties in patients with both total and intact FGF23 measurements. However, in the iron parameter subset, iron deficiency was not associated with higher total FGF23 concentrations, suggesting likely little confounding effect in our cohort. Additionally, other as yet unidentified factors may mediate the inverse association between anemia and total FGF23. In a cohort of adult patients with CKD of similar size to our cohort and with a similar mean eGFR, in multivariable analysis, hemoglobin was inversely associated with total FGF23, independent of iron status (serum ferritin), serum erythropoietin, and inflammation (serum C-reactive protein), as well as eGFR and serum phosphate [27].

The goal of our study was to evaluate how anemia and anemia-related factors affect FGF23 levels; however, a bi-directional relationship may exist in which FGF23 also affects erythropoiesis. Pre-clinical studies have demonstrated that administration of exogenous intact FGF23 protein decreases kidney erythropoietin mRNA expression [26], serum erythropoietin concentrations [43], and erythropoietic parameters [43], whereas administration of an FGF23 blocking peptide increases serum erythropoietin concentrations and some erythropoietic parameters [44], suggesting that FGF23 negatively regulates erythropoiesis. Consistent with these pre-clinical studies, in a large cohort of adult patients with CKD (the Chronic Renal Insufficiency Cohort), increased baseline concentrations of total FGF23 were independently associated with both prevalent and incident anemia [45]. Therefore, whereas iron deficiency, increased erythropoietin, and anemia may contribute to higher FGF23 levels (with total FGF23 increased more so than intact FGF23), elevated FGF23, in turn, may contribute to decreased erythropoietin and impaired erythropoiesis.

Other cohort studies of pediatric patients with CKD have evaluated associations between hemoglobin concentrations and FGF23 levels [46–48]. One cross-sectional study observed that hemoglobin was inversely associated with total FGF23, but was not associated with intact FGF23 [46], similar to our findings. Another study also found that hemoglobin was inversely associated with total FGF23 [47], while a third study observed that hemoglobin was inversely associated with intact FGF23 [48]. However, all three of these cohorts were relatively small (53–62 patients), limiting the number of covariables for which the results could be adjusted; the former two studies included patients with kidney transplants or on dialysis, respectively; and the latter two studies used different FGF23 assays than those used in the present study.

Our study has strengths and limitations. To our knowledge, it is the largest study to date to evaluate associations among anemia, iron parameters, and FGF23 in a pediatric CKD cohort. In our multiple linear regression modeling, we were able to adjust for a large number of potentially confounding covariables. Additionally, our study benefits from the concurrent measurements of total and intact FGF23 concentrations, at least in a subset of study subjects, allowing for insight into possible effects on FGF23 cleavage. Study limitations include incomplete data for all subjects, such that iron parameters and intact FGF23 concentrations were only available in subsets of the entire cohort. Also, CRP may not be a very sensitive marker for inflammation. Lastly, our study is cross-sectional in nature and thus does not provide longitudinal insight as to whether anemia is associated with changes in FGF23 over time.

In summary, our study evaluated associations among anemia, iron parameters, and FGF23 in a large cohort of pediatric patients with CKD, demonstrating independent associations between anemia and higher total FGF23, but not intact FGF23, suggesting possible effects on both FGF23 production and cleavage. Our study highlights important associations between the anemia of CKD and CKD-MBD, and suggests that further studies are warranted to investigate how non-mineral factors influence FGF23 production and metabolism in CKD, and how the treatment of anemia in CKD may affect FGF23 and FGF23-related outcomes.

### Supplementary Information

Below is the link to the electronic supplementary material.

Graphical abstract (PPTX 46 KB)

Supplementary file2 (DOCX 131 KB)
