# Supplementary material for: Associations between anemia and FGF23 in the CKiD study
Source: Pediatr Nephrol. Author manuscript; Available in PMC 2024 Mar 1. (PMC10817837; doi:10.1007/s00467-023-06160-0)
Supplement: 1 [file NIHMS1934073-supplement-1.pdf]

Associations between anemia and FGF23 in the CKiD study

Thomas E, Klomhaus AM, Laster ML, Furth SL, Warady BA, Salusky IB, Hanudel MR

Supplemental Material:

Supplemental Table 1. Definitions of anemia based on the 2012 KDIGO guidelines for pediatric patients with CKD.

Supplemental Figure 1. The total fibroblast growth factor 23 (FGF23) and intact FGF23 enzyme-linked immunosorbent assays (ELISA). Whereas the total FGF23 ELISA detects both the full-length, intact protein and its C-terminal proteolytic fragments, the intact FGF23 assay detects only the full-length form of the hormone. Adapted from Hanudel MR, et al. PLoS One. 2019 Sep 5;14(9):e0222065.

Supplemental Table 2. C-terminal (total) fibroblast growth factor 23 concentrations (RU/ml), stratified by the absence vs. presence of anemia and CKD stage or eGFR quartile.

Supplemental Table 3. C-terminal (total) fibroblast growth factor 23 concentrations (RU/ml), stratified by the absence vs. presence of iron deficiency and CKD stage or eGFR quartile.

Supplemental Table 4. Multivariable linear regression modeling of determinants of log-transformed C-terminal (total) fibroblast growth factor 23 (Table 5, Model 5, n=459).

Supplemental Table 5. Multivariable linear regression modeling of determinants of log-transformed C-terminal (total) fibroblast growth factor 23 in the subset of subjects with iron parameters (n=177 in the fully adjusted model).

Supplemental Table 6. Associations between anemia and log-transformed intact fibroblast growth factor 23.

Supplemental Table 7. Associations between anemia and log-transformed C-terminal (total) or intact fibroblast growth factor 23 in subjects with measurements of both parameters.

<u>Supplemental Table 1</u>. Definitions of anemia based on the 2012 KDIGO guidelines for pediatric patients with CKD.

Age (years)	Hemoglobin (g/dl)
0.5-5	<11.0
5-12	<11.5
12-15	<12.0
>15 female	<12.0
>15 male	<13.0

Supplemental Figure 1. The total fibroblast growth factor 23 (FGF23) and intact FGF23 enzyme-linked immunosorbent assays (ELISA). Whereas the total FGF23 ELISA detects both the full-length, intact protein and its C-terminal proteolytic fragments, the intact FGF23 assay detects only the full-length form of the hormone. Adapted from Hanudel MR, et al. PLoS One. 2019 Sep 5;14(9):e0222065.

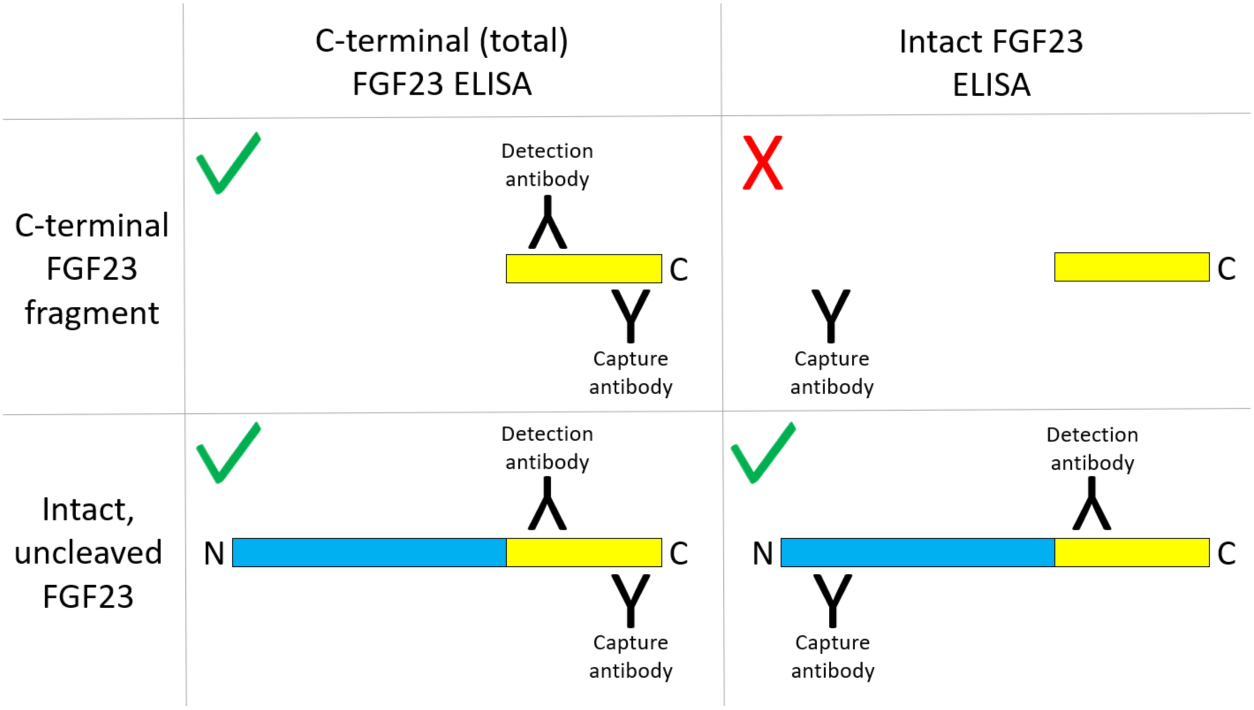

Supplemental Table 2. C-terminal (total) fibroblast growth factor 23 concentrations (RU/ml), stratified by the absence vs. presence of anemia and CKD stage or eGFR quartile.

**CKD Stage 1 (eGFR ≥90 ml/min/1.73m^2) (n=17, 3%)**			**CKD Stage 2 (eGFR 60-89 ml/min/1.73m^2) (n=117, 24%)**			**CKD Stage 3a (eGFR 45-59 ml/min/1.73m^2) (n=145, 29%)**			**CKD Stage 3b (eGFR 30-44 ml/min/1.73m^2) (n=132, 27%)**			**CKD Stage 4 (eGFR 15-29 ml/min/1.73m^2) (n=73, 15%)**			**CKD Stage 5 (eGFR <15 ml/min/1.73m^2) (n=8, 2%)**		
No Anemia (n=13, 76%)	Anemia (n=4, 24%)	P-value	No Anemia (n=111, 95%)	Anemia (n=6, 5%)	P-value	No Anemia (n=130, 90%)	Anemia (n=15, 10%)	P-value	No Anemia (n=96, 73%)	Anemia (n=36, 27%)	P-value	No Anemia (n=38, 52%)	Anemia (n=35, 48%)	P-value	No Anemia (n=1, 13%)	Anemia (n=7, 88%)	P-value
78 (56, 139)	102 (66, 162)	0.62	82 (68, 112)	205 (79, 300)	0.035	101 (78, 147)	139 (93, 407)	0.013	134 (100, 196)	158 (106, 229)	0.26	246 (178, 409)	292 (187, 492)	0.24	335	1005 (394, 2454)	n/a

**eGFR Quartile 1 (61-120 ml/min/1.73m^2) (n=123, 25%)**			**eGFR Quartile 2 (48-60 ml/min/1.73m^2) (n=123, 25%)**			**eGFR Quartile 3 (35-47 ml/min/1.73m^2) (n=123, 25%)**			**eGFR Quartile 4 (7-34 ml/min/1.73m^2) (n=123, 25%)**		
No Anemia (n=115, 93%)	Anemia (n=8, 7%)	P-value	No Anemia (n=112, 91%)	Anemia (n=11, 9%)	P-value	No Anemia (n=100, 81%)	Anemia (n=23, 19%)	P-value	No Anemia (n=62, 50%)	Anemia (n=61, 50%)	P-value
79 (67, 112)	105 (66, 166)	0.25	99 (77, 130)	128 (93, 272)	0.046	126 (97, 185)	148 (91, 232)	0.42	217 (134, 308)	274 (173, 541)	0.017

Data presented as numbers and percentages, or as medians and interquartile ranges. CKD: chronic kidney disease, eGFR: estimated glomerular filtration rate, RU: relative units.

Supplemental Table 3. C-terminal (total) fibroblast growth factor 23 concentrations (RU/ml), stratified by the absence vs. presence of iron deficiency and CKD stage or eGFR quartile.

CKD Stage 1 (eGFR ≥90 ml/min/1.73m²) (n=1, 0%)			CKD Stage 2 (eGFR 60-89 ml/min/1.73m²) (n=35, 18%)			CKD Stage 3a (eGFR 45-59 ml/min/1.73m²) (n=63, 33%)			CKD Stage 3b (eGFR 30-44 ml/min/1.73m²) (n=62, 32%)			CKD Stage 4 (eGFR 15-29 ml/min/1.73m²) (n=27, 14%)			CKD Stage 5 (eGFR <15 ml/min/1.73m²) (n=3, 2%)		
No ID (n=0, 0%)	ID (n=1, 100%)	P-value	No ID (n=24, 69%)	ID (n=11, 31%)	P-value	No ID (n=42, 67%)	ID (n=21, 33%)	P-value	No ID (n=38, 61%)	ID (n=24, 39%)	P-value	No ID (n=18, 67%)	ID (n=9, 33%)	P-value	No ID (n=2, 67%)	ID (n=1, 33%)	P-value
n/a	43	n/a	89 (76, 112)	103 (75, 184)	0.25	110 (86, 160)	133 (108, 196)	0.11	138 (104, 195)	133 (90, 206)	0.51	285 (207, 458)	203 (137, 844)	0.62	1424 (394, 2454)	1225	n/a

eGFR Quartile 1 (56-93 ml/min/1.73m²) (n=48, 25%)			eGFR Quartile 2 (46-55 ml/min/1.73m²) (n=48, 25%)			eGFR Quartile 3 (34-45 ml/min/1.73m²) (n=48, 25%)			eGFR Quartile 4 (7-33 ml/min/1.73m²) (n=47, 25%)		
No ID (n=32, 67%)	ID (n=16, 33%)	P-value	No ID (n=33, 69%)	ID (n=15, 31%)	P-value	No ID (n=27, 56%)	ID (n=21, 44%)	P-value	No ID (n=32, 68%)	ID (n=15, 32%)	P-value
88 (76, 110)	111 (78, 177)	0.13	114 (89, 168)	133 (97, 207)	0.37	132 (104, 184)	143 (90, 215)	0.99	235 (160, 436)	177 (115, 531)	0.36

Data presented as numbers and percentages, or as medians and interquartile ranges. CKD: chronic kidney disease, eGFR: estimated glomerular filtration rate, ID: iron deficiency, RU: relative units.

Supplemental Table 4. Multivariable linear regression modeling of determinants of log-transformed C-terminal (total) fibroblast growth factor 23 (Table 5, Model 5, n=459).

Parameter	Standardized β (95% CI)	P-value	VIF
Age (years)	0.02 (−0.02, 0.05)	0.39	2.61
Male sex	−0.03 (−0.08, 0.02)	0.19	1.07
Race: African American	−0.001 (−0.07, 0.07)	0.97	1.17
Race: Asian	0.11 (−0.04, 0.26)	0.14	1.05
Race: Native American	0.04 (−0.14, 0.23)	0.63	1.10
Race: Other	0.05 (−0.08, 0.18)	0.44	1.21
Race: Multiple	−0.04 (−0.12, 0.04)	0.33	1.05
Hispanic ethnicity	−0.08 (−0.16, −0.01)	0.030	1.16
CKD duration (years)	−0.03 (−0.07, 0.004)	0.08	2.58
Glomerular disease	0.04 (−0.05, 0.12)	0.39	2.26
eGFR (ml/min/1.73m^2)	−0.11 (−0.14, −0.08)	<0.001	1.83
C-reactive protein (mg/l)	0.01 (−0.01, 0.04)	0.30	1.07
ESA use	0.08 (−0.01, 0.17)	0.08	1.44
Iron supplementation	−0.07 (−0.13, −0.01)	0.023	1.38
Calcium (mg/dl)	0.01 (−0.02, 0.03)	0.55	1.38
Phosphate SDS for age	0.05 (0.02, 0.07)	<0.001	1.18
Parathyroid hormone (pg/ml)	0.03 (0.01, 0.06)	0.018	1.46
Phosphate binder use	0.02 (−0.05, 0.09)	0.31	1.20
Native vitamin D (25D) use	−0.05 (−0.12, 0.02)	0.18	1.21
Active vitamin D (1,25D) use	0.03 (−0.03, 0.09)	0.31	1.39
Anemia	0.10 (0.04, 0.17)	0.002	1.35

CI: confidence interval, CKD: chronic kidney disease, eGFR: estimated glomerular filtration rate, ESA: erythropoiesis-stimulating agent, SDS: standard deviation score, VIF: variance inflation factor.

Supplemental Table 5. Multivariable linear regression modeling of determinants of log-transformed C-terminal (total) fibroblast growth factor 23 in the subset of subjects with iron parameters (n=177 in the fully adjusted model).

Parameter	Standardized β (95% CI)	P-value	VIF
Age (years)	0.02 (−0.05, 0.08)	0.60	2.99
Male sex	−0.08 (−0.16, 0.01)	0.07	1.19
Race: African American	−0.03 (−0.14, 0.09)	0.44	1.29
Race: Asian	0.14 (−0.09, 0.38)	0.23	1.14
Race: Native American	0.03 (−0.30, 0.37)	0.85	1.38
Race: Other	0.05 (−0.23, 0.33)	0.73	1.29
Race: Multiple	−0.08 (−0.20, 0.04)	0.20	1.09
Hispanic ethnicity	−0.22 (−0.37, −0.06)	0.007	1.32
CKD duration (years)	−0.03 (−0.10, 0.03)	0.32	2.96
Glomerular disease	0.01 (−0.13, 0.16)	0.86	2.35
eGFR (ml/min/1.73m^2)	−0.14 (−0.20, −0.08)	<0.001	1.93
C-reactive protein (mg/l)	0.01 (−0.02, 0.05)	0.43	1.23
ESA use	0.002 (−0.14, 0.15)	0.98	1.65
Iron supplementation	−0.08 (−0.18, 0.01)	0.09	1.46
Calcium (mg/dl)	0.03 (−0.02, 0.08)	0.19	1.46
Phosphate SDS for age	0.03 (−0.02, 0.08)	0.21	1.35
Parathyroid hormone (pg/ml)	0.05 (0.003, 0.09)	0.037	1.50
Phosphate binder use	−0.001 (−0.12, 0.12)	0.98	1.38
Native vitamin D (25D) use	−0.07 (−0.22, 0.08)	0.37	1.44
Active vitamin D (1,25D) use	0.02 (−0.07, 0.11)	0.61	1.38
Iron deficiency	0.05 (−0.04, 0.13)	0.26	1.21
Anemia	0.10 (−0.02, 0.21)	0.09	1.70

CI: confidence interval, CKD: chronic kidney disease, eGFR: estimated glomerular filtration rate, ESA: erythropoiesis-stimulating agent, SDS: standard deviation score, VIF: variance inflation factor.

Supplemental Table 6. Associations between anemia and log-transformed intact fibroblast growth factor 23.

Model	Covariables	Number of Subjects	Standardized β (95% CI)	P-value
1	Unadjusted	185	0.16 (0.04, 0.28)	0.011
2	Model 1 + adjustment for age, sex, race, and ethnicity	185	0.14 (0.02, 0.26)	0.025
3	Model 2 + adjustment for CKD duration, glomerular disease etiology, and eGFR	180	0.07 (−0.06, 0.20)	0.29
4	Model 3 + adjustment for CRP, ESA use, and iron supplementation	178	0.07 (−0.06, 0.20)	0.28
5	Model 4 + adjustment for calcium, phosphate SDS for age, PTH, phosphate binder use, 25D use, and 1,25D use	176	0.02 (−0.12, 0.15)	0.81

CI: confidence interval, CKD: chronic kidney disease, CRP: C-reactive protein, eGFR: estimated glomerular filtration rate, ESA: erythropoiesis-stimulating agent, PTH: parathyroid hormone, SDS: standard deviation score.

<u>Supplemental Table 7</u>. Associations between anemia and log-transformed C-terminal (total) or intact fibroblast growth factor 23 in subjects with measurements of both parameters.

Model	Covariables	Number of Subjects	DV: Log tFGF23 Standardized β (95% CI)	DV: Log iFGF23 Standardized β (95% CI)
1	Unadjusted	185	0.27 (0.17, 0.37) p<0.001	0.16 (0.04, 0.28) p=0.011
2	Model 1 + adjustment for age, sex, race, and ethnicity	185	0.26 (0.15, 0.37) p<0.001	0.14 (0.02, 0.26) p=0.025
3	Model 2 + adjustment for CKD duration, glomerular disease etiology, and eGFR	180	0.17 (0.06, 0.28) p=0.004	0.07 (−0.06, 0.20) p=0.29
4	Model 3 + adjustment for CRP, ESA use, and iron supplementation	178	0.18 (0.07, 0.29) p=0.001	0.07 (−0.06, 0.20) p=0.28
5	Model 4 + adjustment for calcium, phosphate SDS for age, PTH, phosphate binder use, 25D use and 1,25D use	176	0.16 (0.04, 0.27) p=0.008	0.02 (−0.12, 0.15) p=0.81

CI: confidence interval, CKD: chronic kidney disease, CRP: C-reactive protein, DV: dependent variable, eGFR: estimated glomerular filtration rate, ESA: erythropoiesis-stimulating agent, iFGF23: intact fibroblast growth factor 23, PTH: parathyroid hormone, SDS: standard deviation score, tFGF23: total fibroblast growth factor 23.
