# Supplementary material for: Associations between anemia and FGF23 in the CKiD study
Source: Pediatr Nephrol. 2023 Sep 26;39(3):837–47. doi: 10.1007/s00467-023-06160-0 (PMC10817837; doi:10.1007/s00467-023-06160-0)
Supplement: Supplementary file 1 [file 467_2023_6160_MOESM2_ESM.docx]

**Associations between anemia and FGF23 in the CKiD study**

Thomas E, Klomhaus AM, Laster ML, Furth SL, Warady BA, Salusky IB, Hanudel MR

**Supplemental Material:**

**Supplemental Table 1.** Definitions of anemia based on the 2012 KDIGO guidelines for pediatric patients with CKD.

**Supplemental Figure 1.** The total fibroblast growth factor 23 (FGF23) and intact FGF23 enzyme-linked immunosorbent assays (ELISA). Whereas the total FGF23 ELISA detects both the full-length, intact protein and its C-terminal proteolytic fragments, the intact FGF23 assay detects only the full-length form of the hormone. Adapted from Hanudel MR, et al. PLoS One. 2019 Sep 5;14(9):e0222065.

**Supplemental Table 2.** C-terminal (total) fibroblast growth factor 23 concentrations (RU/ml), stratified by the absence vs. presence of anemia and CKD stage or eGFR quartile.

**Supplemental Table 3.** C-terminal (total) fibroblast growth factor 23 concentrations (RU/ml), stratified by the absence vs. presence of iron deficiency and CKD stage or eGFR quartile.

**Supplemental Table 4.** Multivariable linear regression modeling of determinants of log-transformed C-terminal (total) fibroblast growth factor 23 (Table 5, Model 5, n=459).

**Supplemental Table 5.** Multivariable linear regression modeling of determinants of log-transformed C-terminal (total) fibroblast growth factor 23 in the subset of subjects with iron parameters (n=177 in the fully adjusted model).

**Supplemental Table 6.** Associations between anemia and log-transformed intact fibroblast growth factor 23.

**Supplemental Table 7.** Associations between anemia and log-transformed C-terminal (total) or intact fibroblast growth factor 23 in subjects with measurements of both parameters.

**Supplemental Table 1.** Definitions of anemia based on the 2012 KDIGO guidelines for pediatric patients with CKD.

| **Age (years)** | **Hemoglobin (g/dl)** |
| --- | --- |
| 0.5-5 | <11.0 |
| 5-12 | <11.5 |
| 12-15 | <12.0 |
| >15 female | <12.0 |
| >15 male | <13.0 |

**Supplemental Figure 1.** The total fibroblast growth factor 23 (FGF23) and intact FGF23 enzyme-linked immunosorbent assays (ELISA). Whereas the total FGF23 ELISA detects both the full-length, intact protein and its C-terminal proteolytic fragments, the intact FGF23 assay detects only the full-length form of the hormone. Adapted from Hanudel MR, et al. PLoS One. 2019 Sep 5;14(9):e0222065.

**Supplemental Table 2.** C-terminal (total) fibroblast growth factor 23 concentrations (RU/ml), stratified by the absence vs. presence of anemia and CKD stage or eGFR quartile.

| **CKD Stage 1** **(eGFR ≥90 ml/min/1.73m**^**2**^**)** **(n=17, 3%)** |  |  |  | **CKD Stage 2** **(eGFR 60-89 ml/min/1.73m**^**2**^**)** **(n=117, 24%)** |  |  |  |  | **CKD Stage 3a** **(eGFR 45-59 ml/min/1.73m**^**2**^**)** **(n=145, 29%)** |  |  |  |  | **CKD Stage 3b** **(eGFR 30-44 ml/min/1.73m**^**2**^**)** **(n=132, 27%)** |  |  |  | **CKD Stage 4** **(eGFR 15-29 ml/min/1.73m**^**2**^**)** **(n=73, 15%)** |  |  |  |  | **CKD Stage 5** **(eGFR <15 ml/min/1.73m**^**2**^**)** **(n=8, 2%)** |  |  |  |  |
| --- | --- | --- | --- | --- | --- | --- | --- | --- | --- | --- | --- | --- | --- | --- | --- | --- | --- | --- | --- | --- | --- | --- | --- | --- | --- | --- | --- |
| No Anemia (n=13, 76%) | Anemia (n=4, 24%) |  | P-value | No Anemia (n=111, 95%) |  | Anemia (n=6, 5%) |  | P-value | No Anemia (n=130, 90%) |  | Anemia (n=15, 10%) |  | P-value | No Anemia (n=96, 73%) | Anemia (n=36, 27%) |  | P-value | No Anemia (n=38, 52%) |  | Anemia (n=35, 48%) |  | P-value | No Anemia (n=1, 13%) |  | Anemia (n=7, 88%) |  | P-value |
| 78 (56, 139) | 102 (66, 162) |  | 0.62 | 82 (68, 112) |  | 205 (79, 300) |  | 0.035 | 101 (78, 147) |  | 139 (93, 407) |  | 0.013 | 134 (100, 196) | 158 (106, 229) |  | 0.26 | 246 (178, 409) |  | 292 (187, 492) |  | 0.24 | 335 |  | 1005 (394, 2454) |  | n/a |
|  |  |  |  |  |  |  |  |  |  |  |  |  |  |  |  |  |  |  |  |  |  |  |  |  |  |  |  |
| **eGFR Quartile 1** **(61-120 ml/min/1.73m**^**2**^**)** **(n=123, 25%)** |  |  |  |  |  |  | **eGFR Quartile 2** **(48-60 ml/min/1.73m**^**2**^**)** **(n=123, 25%)** |  |  |  |  |  |  | **eGFR Quartile 3** **(35-47 ml/min/1.73m**^**2**^**)** **(n=123, 25%)** |  |  |  |  |  |  | **eGFR Quartile 4** **(7-34 ml/min/1.73m**^**2**^**)** **(n=123, 25%)** |  |  |  |  |  |  |
| No Anemia (n=115, 93%) |  | Anemia (n=8, 7%) |  |  | P-value |  | No Anemia (n=112, 91%) |  |  | Anemia (n=11, 9%) |  | P-value |  | No Anemia (n=100, 81%) |  | Anemia (n=23, 19%) |  |  | P-value |  | No Anemia (n=62, 50%) |  |  | Anemia (n=61, 50%) |  | P-value |  |
| 79 (67, 112) |  | 105 (66, 166) |  |  | 0.25 |  | 99 (77, 130) |  |  | 128 (93, 272) |  | 0.046 |  | 126 (97, 185) |  | 148 (91, 232) |  |  | 0.42 |  | 217 (134, 308) |  |  | 274 (173, 541) |  | 0.017 |  |
|  |  |  |  |  |  |  |  |  |  |  |  |  |  |  |  |  |  |  |  |  |  |  |  |  |  |  |  |

Data presented as numbers and percentages, or as medians and interquartile ranges. CKD: chronic kidney disease, eGFR: estimated glomerular filtration rate, RU: relative units.

**Supplemental Table 3.** C-terminal (total) fibroblast growth factor 23 concentrations (RU/ml), stratified by the absence vs. presence of iron deficiency and CKD stage or eGFR quartile.

| **CKD Stage 1** **(eGFR ≥90 ml/min/1.73m**^**2**^**)** **(n=1, 0%)** |  |  |  | **CKD Stage 2** **(eGFR 60-89 ml/min/1.73m**^**2**^**)** **(n=35, 18%)** |  |  |  |  | **CKD Stage 3a** **(eGFR 45-59 ml/min/1.73m**^**2**^**)** **(n=63, 33%)** |  |  |  |  | **CKD Stage 3b** **(eGFR 30-44 ml/min/1.73m**^**2**^**)** **(n=62, 32%)** |  |  |  | **CKD Stage 4** **(eGFR 15-29 ml/min/1.73m**^**2**^**)** **(n=27, 14%)** |  |  |  |  | **CKD Stage 5** **(eGFR <15 ml/min/1.73m**^**2**^**)** **(n=3, 2%)** |  |  |  |  |
| --- | --- | --- | --- | --- | --- | --- | --- | --- | --- | --- | --- | --- | --- | --- | --- | --- | --- | --- | --- | --- | --- | --- | --- | --- | --- | --- | --- |
| No ID (n=0, 0%) | ID (n=1, 100%) |  | P-value | No ID (n=24, 69%) |  | ID (n=11, 31%) |  | P-value | No ID (n=42, 67%) |  | ID (n=21, 33%) |  | P-value | No ID (n=38, 61%) | ID (n=24, 39%) |  | P-value | No ID (n=18, 67%) |  | ID (n=9, 33%) |  | P-value | No ID (n=2, 67%) |  | ID (n=1, 33%) |  | P-value |
| n/a | 43 |  | n/a | 89 (76, 112) |  | 103 (75, 184) |  | 0.25 | 110 (86, 160) |  | 133 (108, 196) |  | 0.11 | 138 (104, 195) | 133 (90, 206) |  | 0.51 | 285 (207, 458) |  | 203 (137, 844) |  | 0.62 | 1424 (394, 2454) |  | 1225 |  | n/a |
|  |  |  |  |  |  |  |  |  |  |  |  |  |  |  |  |  |  |  |  |  |  |  |  |  |  |  |  |
| **eGFR Quartile 1** **(56-93 ml/min/1.73m**^**2**^**)** **(n=48, 25%)** |  |  |  |  |  |  | **eGFR Quartile 2** **(46-55 ml/min/1.73m**^**2**^**)** **(n=48, 25%)** |  |  |  |  |  |  | **eGFR Quartile 3** **(34-45 ml/min/1.73m**^**2**^**)** **(n=48, 25%)** |  |  |  |  |  |  | **eGFR Quartile 4** **(7-33 ml/min/1.73m**^**2**^**)** **(n=47, 25%)** |  |  |  |  |  |  |
| No ID (n=32, 67%) |  | ID (n=16, 33%) |  |  | P-value |  | No ID (n=33, 69%) |  |  | ID (n=15, 31%) |  | P-value |  | No ID (n=27, 56%) |  | ID (n=21, 44%) |  |  | P-value |  | No ID (n=32, 68%) |  |  | ID (n=15, 32%) |  | P-value |  |
| 88 (76, 110) |  | 111 (78, 177) |  |  | 0.13 |  | 114 (89, 168) |  |  | 133 (97, 207) |  | 0.37 |  | 132 (104, 184) |  | 143 (90, 215) |  |  | 0.99 |  | 235 (160, 436) |  |  | 177 (115, 531) |  | 0.36 |  |
|  |  |  |  |  |  |  |  |  |  |  |  |  |  |  |  |  |  |  |  |  |  |  |  |  |  |  |  |

Data presented as numbers and percentages, or as medians and interquartile ranges. CKD: chronic kidney disease, eGFR: estimated glomerular filtration rate, ID: iron deficiency, RU: relative units.

**Supplemental Table 4.** Multivariable linear regression modeling of determinants of log-transformed C-terminal (total) fibroblast growth factor 23 (Table 5, Model 5, n=459).

| **Parameter** | **Standardized β (95% CI)** | **P-value** | **VIF** |
| --- | --- | --- | --- |
| Age (years) | 0.02 (−0.02, 0.05) | 0.39 | 2.61 |
| Male sex | −0.03 (−0.08, 0.02) | 0.19 | 1.07 |
| Race: African American | −0.001 (−0.07, 0.07) | 0.97 | 1.17 |
| Race: Asian | 0.11 (−0.04, 0.26) | 0.14 | 1.05 |
| Race: Native American | 0.04 (−0.14, 0.23) | 0.63 | 1.10 |
| Race: Other | 0.05 (−0.08, 0.18) | 0.44 | 1.21 |
| Race: Multiple | −0.04 (−0.12, 0.04) | 0.33 | 1.05 |
| Hispanic ethnicity | −0.08 (−0.16, −0.01) | 0.030 | 1.16 |
| CKD duration (years) | −0.03 (−0.07, 0.004) | 0.08 | 2.58 |
| Glomerular disease | 0.04 (−0.05, 0.12) | 0.39 | 2.26 |
| eGFR (ml/min/1.73m^2^) | −0.11 (−0.14, −0.08) | <0.001 | 1.83 |
| C-reactive protein (mg/l) | 0.01 (−0.01, 0.04) | 0.30 | 1.07 |
| ESA use | 0.08 (−0.01, 0.17) | 0.08 | 1.44 |
| Iron supplementation | −0.07 (−0.13, −0.01) | 0.023 | 1.38 |
| Calcium (mg/dl) | 0.01 (−0.02, 0.03) | 0.55 | 1.38 |
| Phosphate SDS for age | 0.05 (0.02, 0.07) | <0.001 | 1.18 |
| Parathyroid hormone (pg/ml) | 0.03 (0.01, 0.06) | 0.018 | 1.46 |
| Phosphate binder use | 0.02 (−0.05, 0.09) | 0.31 | 1.20 |
| Native vitamin D (25D) use | −0.05 (−0.12, 0.02) | 0.18 | 1.21 |
| Active vitamin D (1,25D) use | 0.03 (−0.03, 0.09) | 0.31 | 1.39 |
| Anemia | 0.10 (0.04, 0.17) | 0.002 | 1.35 |

CI: confidence interval, CKD: chronic kidney disease, eGFR: estimated glomerular filtration rate, ESA: erythropoiesis-stimulating agent, SDS: standard deviation score, VIF: variance inflation factor.

**Supplemental Table 5.** Multivariable linear regression modeling of determinants of log-transformed C-terminal (total) fibroblast growth factor 23 in the subset of subjects with iron parameters (n=177 in the fully adjusted model).

| **Parameter** | **Standardized β (95% CI)** | **P-value** | **VIF** |
| --- | --- | --- | --- |
| Age (years) | 0.02 (−0.05, 0.08) | 0.60 | 2.99 |
| Male sex | −0.08 (−0.16, 0.01) | 0.07 | 1.19 |
| Race: African American | −0.03 (−0.14, 0.09) | 0.44 | 1.29 |
| Race: Asian | 0.14 (−0.09, 0.38) | 0.23 | 1.14 |
| Race: Native American | 0.03 (−0.30, 0.37) | 0.85 | 1.38 |
| Race: Other | 0.05 (−0.23, 0.33) | 0.73 | 1.29 |
| Race: Multiple | −0.08 (−0.20, 0.04) | 0.20 | 1.09 |
| Hispanic ethnicity | −0.22 (−0.37, −0.06) | 0.007 | 1.32 |
| CKD duration (years) | −0.03 (−0.10, 0.03) | 0.32 | 2.96 |
| Glomerular disease | 0.01 (−0.13, 0.16) | 0.86 | 2.35 |
| eGFR (ml/min/1.73m^2^) | −0.14 (−0.20, −0.08) | <0.001 | 1.93 |
| C-reactive protein (mg/l) | 0.01 (−0.02, 0.05) | 0.43 | 1.23 |
| ESA use | 0.002 (−0.14, 0.15) | 0.98 | 1.65 |
| Iron supplementation | −0.08 (−0.18, 0.01) | 0.09 | 1.46 |
| Calcium (mg/dl) | 0.03 (−0.02, 0.08) | 0.19 | 1.46 |
| Phosphate SDS for age | 0.03 (−0.02, 0.08) | 0.21 | 1.35 |
| Parathyroid hormone (pg/ml) | 0.05 (0.003, 0.09) | 0.037 | 1.50 |
| Phosphate binder use | −0.001 (−0.12, 0.12) | 0.98 | 1.38 |
| Native vitamin D (25D) use | −0.07 (−0.22, 0.08) | 0.37 | 1.44 |
| Active vitamin D (1,25D) use | 0.02 (−0.07, 0.11) | 0.61 | 1.38 |
| Iron deficiency | 0.05 (−0.04, 0.13) | 0.26 | 1.21 |
| Anemia | 0.10 (−0.02, 0.21) | 0.09 | 1.70 |

CI: confidence interval, CKD: chronic kidney disease, eGFR: estimated glomerular filtration rate, ESA: erythropoiesis-stimulating agent, SDS: standard deviation score, VIF: variance inflation factor.

**Supplemental Table 6.** Associations between anemia and log-transformed intact fibroblast growth factor 23.

| **Model** | **Covariables** | **Number of Subjects** | **Standardized β** **(95% CI)** | **P-value** |
| --- | --- | --- | --- | --- |
| 1 | Unadjusted | 185 | 0.16 (0.04, 0.28) | 0.011 |
| 2 | Model 1 + adjustment for age, sex, race, and ethnicity | 185 | 0.14 (0.02, 0.26) | 0.025 |
| 3 | Model 2 + adjustment for CKD duration, glomerular disease etiology, and eGFR | 180 | 0.07 (−0.06, 0.20) | 0.29 |
| 4 | Model 3 + adjustment for CRP, ESA use, and iron supplementation | 178 | 0.07 (−0.06, 0.20) | 0.28 |
| 5 | Model 4 + adjustment for calcium, phosphate SDS for age, PTH, phosphate binder use, 25D use, and 1,25D use | 176 | 0.02 (−0.12, 0.15) | 0.81 |

CI: confidence interval, CKD: chronic kidney disease, CRP: C-reactive protein, eGFR: estimated glomerular filtration rate, ESA: erythropoiesis-stimulating agent, PTH: parathyroid hormone, SDS: standard deviation score.

**Supplemental Table 7.** Associations between anemia and log-transformed C-terminal (total) or intact fibroblast growth factor 23 in subjects with measurements of both parameters.

| **Model** | **Covariables** | **Number of Subjects** | **DV: Log tFGF23** **Standardized β** **(95% CI)** | **DV: Log iFGF23** **Standardized β** **(95% CI)** |
| --- | --- | --- | --- | --- |
| 1 | Unadjusted | 185 | 0.27 (0.17, 0.37) p<0.001 | 0.16 (0.04, 0.28) p=0.011 |
| 2 | Model 1 + adjustment for age, sex, race, and ethnicity | 185 | 0.26 (0.15, 0.37) p<0.001 | 0.14 (0.02, 0.26) p=0.025 |
| 3 | Model 2 + adjustment for CKD duration, glomerular disease etiology, and eGFR | 180 | 0.17 (0.06, 0.28) p=0.004 | 0.07 (−0.06, 0.20) p=0.29 |
| 4 | Model 3 + adjustment for CRP, ESA use, and iron supplementation | 178 | 0.18 (0.07, 0.29) p=0.001 | 0.07 (−0.06, 0.20) p=0.28 |
| 5 | Model 4 + adjustment for calcium, phosphate SDS for age, PTH, phosphate binder use, 25D use and 1,25D use | 176 | 0.16 (0.04, 0.27) p=0.008 | 0.02 (−0.12, 0.15) p=0.81 |

CI: confidence interval, CKD: chronic kidney disease, CRP: C-reactive protein, DV: dependent variable, eGFR: estimated glomerular filtration rate, ESA: erythropoiesis-stimulating agent, iFGF23: intact fibroblast growth factor 23, PTH: parathyroid hormone, SDS: standard deviation score, tFGF23: total fibroblast growth factor 23.
